# Retrospective Diagnosis of Parkinsonian Syndromes Using Whole-Brain Atrophy Rates

**DOI:** 10.3389/fnagi.2017.00099

**Published:** 2017-04-19

**Authors:** Carlos Guevara, Kateryna Bulatova, Wendy Soruco, Guido Gonzalez, Gonzalo A. Farías

**Affiliations:** Facultad de Medicina, Universidad de ChileSantiago, Chile

**Keywords:** whole brain atrophy rate, multiple system atrophy, progressive supranuclear palsy, idiopathic Parkinson's disease

## Abstract

**Objective:** The absence of markers for *ante-mortem* diagnosis of idiopathic Parkinson's disease (IPD), multiple system atrophy (MSA), and progressive supranuclear palsy (PSP) results in these disorders being commonly mistaken for each other, particularly in the initial stages. We aimed to investigate annualized whole-brain atrophy rates (a-WBAR) in these disorders to aid in the diagnosis between IPD vs. PSP and MSA.

**Methods:** Ten healthy controls, 20 IPD, 39 PSP, and 41 MSA patients were studied using Structural Imaging Evaluation with Normalization of Atrophy (SIENA). SIENA is an MRI-based algorithm that quantifies brain tissue volume and does not require radiotracers. SIENA has been shown to have a low estimation error for atrophy rate over the whole brain (0.5%).

**Results:** In controls, the a-WBAR was 0.37% ± 0.28 (CI 95% 0.17–0.57), while in IPD a-WBAR was 0.54% ± 0.38 (CI 95% 0.32–0.68). The IPD patients did not differ from the controls. In PSP, the a-WBAR was 1.93% ± 1.1 (CI 95% 1.5–2.2). In MSA a-WBAR was 1.65% ± 0.9 (CI 95%1.37–1.93). MSA did not differ from PSP. The a-WBAR in PSP and MSA were significantly higher than in IPD (*p* < 0.001). a-WBAR 0.6% differentiated patients with IPD from those with PSA and MSA with 91% sensitivity and 80% specificity.

**Conclusions:** a-WBAR within the normal range is unlikely to be observed in PSP or MSA. a-WBAR may add a potential retrospective application to improve the diagnostic accuracy of MSA and PSP vs. IPD during the first year of clinical assessment.

## Introduction

Multiple system atrophy (MSA) and progressive supranuclear palsy (PSP)—sometimes designated as “Parkinson plus syndromes”—are debilitating neurodegenerative disorders with heterogeneous presentation, inexorable progression, and a median survival of between 5 and 10 years. There is a need to improve the differentiation between idiopathic Parkinson's disease (IPD) and MSA vs. PSP. PSP and MSA can be misdiagnosed as IPD (and vice versa), especially in early stages, as these disorders share some common clinical features, such as bradykinesia and rigidity and even initial response to levodopa treatment, making the diagnosis, which is initially based on clinical presentation only, rather uncertain. Indeed, in 2004 Adler et al. reported that only 26% of IPD cases with signs and symptoms present for < 5 years had neuropathologic confirmation (Adler et al., 2014). Although a number of neuroimaging techniques allow for partial distinction among these diseases (Politis, 2014), no neuroimaging modalities are specifically recommended for routine use in clinical practice for the differential diagnosis between IPD vs. MSA and PSP.

Whole brain atrophy rates (WBAR) from magnetic resonance imaging (MRI) data may be an informative way to quantify disease progression in an unbiased fashion. This approach reduces inter- individual variability in brain size and morphology when baseline scans are used as reference point so that the subject acts as his or her own control. This avenue has been extensively explored in Alzheimer disease, (Fox and Freeborough, 1997; Schott et al., 2005; Ridha et al., 2008; Sluimer et al., 2008a,b) and also in other degenerative dementias such as frontotemporal dementia (Chan et al., 2001; Gordon et al., 2010) and Huntington disease (Hobbs et al., 2010). For normal aging, the annualized-WBAR (a-WBAR) has been estimated to be below 0.6% (Josephs et al., 2006; Whitwell et al., 2007; Sluimer et al., 2008a). To date few studies have used such techniques in PSP and MSA with small numbers of patients. In six autopsy-confirmed PSP cases (Josephs et al., 2006), the a-WBAR [measured using the boundary shift integral (BSI; Freeborough et al., 1997), a (semi-) automated technique] was 1.3%. In another five proven PSP cases, this figure was 1%(Josephs et al., 2006; Whitwell et al., 2007); in another study, also using BSI, a-WBAR estimates were approximately 1% for both PSP and MSA based on 17 PSP cases and 9 cases with MSA-P (Paviour et al., 2006).

An alternative method is provided by structural image evaluation, using normalization, of atrophy (SIENA; Smith et al., 2001, 2002; http://www.fmrib.ox.ac.uk/analysis/research/siena). The suitability of SIENA for longitudinal studies is based on: (a) it is direct, based on a registration of two scans taken at different time points, without the confounding effects of choice of a “template” to which to register, (b) all the stages are fully automated; (c) it has been shown to be robust to changes in acquisition parameters including pulse sequence and slice thickness (Smith et al., 2001), which is an important advantage in clinical trials which are usually multi-center. SIENA has been shown to have a low estimation error for atrophy rate over the whole brain (0.5%; Smith et al., 2001, 2002).

In this study, we used SIENA to estimate a-WBAR in IPD, PSP, and MSA. We aimed to explore the retrospective application of a-WBAR to differentiate IPD from MSA and PSP, after 1 year from the baseline assessment and before 5 years of the disease course.

## Materials and methods

### Subjects and clinical assessment

One hundred and ten participants (10 healthy controls, 20 IPD without dementia, 39 PSP, and 41 MSA patients) were recruited from the Movement Disorders Clinic at the Hospital San Juan de Dios, Santiago, Chile. Internationally established operational criteria were used to assess the diagnoses of MSA, PSP, and IPD (Wenning et al., 1997; Hughes et al., 2001; Litvan et al., 2003). Controls were independently functioning community dwellers, did not have active neurologic or psychiatric conditions, did not have cognitive complaints, and had a normal neurological examination. Fourteen IPD patients had the tremor dominant phenotype and six had the postural instability gait disorder phenotype. Of the 39 PSP patients, 30 had the typical features of classic PSP (Richardson's syndrome). Nine patients were clinically classified as having atypical profiles: four with tremor and moderate L-dopa responsiveness (PSP-Parkinsonism variant), three PSP with corticobasal syndrome (PSP-CBS) and two PSP with progressive nofluent aphasia (PSP-PNFA; Respondek et al., 2014). Thirty-five probable MSA patients were categorized as MSA-P (predominant Parkinsonian features) and six as MSA-C (predominant cerebellar features). All participants were assessed on their usual dopaminergic medication and the IPD patients were evaluated in the “on state.” The patients' demographics and clinical variables are presented in Table 1.

**Table 1:** **Baseline demographics, clinical features, and a-WBAR**.

	**Controls *N* = 10**	**IPD *N* = 20**	**PSP *N* = 39**	**MSA *N* = 41**	**Group comparisons**	**Significant pair-wise comparison**
Age (years)^a^	64.6 ± 9.9	62.2 ± 11.5	68.2 ± 6.3	60.4 ± 7.7	*F* = 6.18	PSP vs. MSA < 0.001
Mean ±SD					*df* = 3	
					*p* = 0.01	
Gender (M:F)^b^	3:7	8:12	21:18	32:9	χ^2^ = 13	
					*df* = 3	
					*p* = 0.005	
Disease duration^a^ (years)	N/A	3.1 ± 3.3	3.0 ± 1.7	4.3 ± 2.3	*F* = 3.4	PSP vs. MSA = 0.04
Mean ± SD					*df* = 2	
					*p* = 0.03	
a-WBAR^a^	0.37% ± 0.28	0.54% ± 0.38	1.93% ± 1.1	1.65% ± 0.9	*F* = 16	IPD vs. MSA < 0.001
(Mean ±SD plus 95% confident interval)	(0.17–0.57)	(0.32–0.68)	(1.5–2.2)	(1.3–1.9)	*df* = 3	IPD vs. PSP < 0.001
					*p* < 0.001	
Baseline UPDRS III^c^		23.2 ± 12	31 ± 13	36.1 ± 18	χ^2^ = 7	IPD vs. MSA = 0.01
(median score plus range)		(3–46)	(6–62)	(10–67)	*df* = 2	
					*p* = 0.03	
Annualized UPDRS change		1.18 ± 12.7	5.6 ± 9.5^*^	6.1 ± 6.9^*^		
(Mean ± SD plus range)		(−4.7–7.1)	(2.5–8.7)	(3.9- 8.6)		
H &Y^c^		1.9 ± 0.6	3.0 ± 0.8	3.0 ± 0.9	χ^2^ = 27	IPD vs. MSA < 0.001
(Mean ±SD score plus range)		(1.0–3.0)	(2.0–5.0)	(1.0–5.0)	*df* = 2	IPD vs. PSP < 0.001
					*p* < 0.001	
Annualized H &Y change		0.1 ± 0.6	0.45 ± 0.55^*^	0.4 ± 0.4^*^		
(Mean ± SD plus range)		(−0.15–0.4)	(0.27–0.63)	(0.28–0.6)		
FAB^c^		14.6 ± 3.5	8.9 ± 4.2	14.0 ± 3.3	χ^2^ = 19	IPD vs. PSP < 0.001
		(5–18)	(0–16)	(4–18)	*df* = 2	MSA vs. PSP < 0.001
					*p* = 0.016	
Annualized FAB change (Mean ±SD plus range)		−0.1 ± 1.9 (−1.0–0.8)	−0.46 ± 3.6 ^*^(−1.8–0.9)	−0.6 ± 2.08^*^ (−1–0.17)		

a *ANOVA test*.

b *Chi square test*.

c *Kruskal-Wallis test and post hoc procedure with MannWhitney test p = 0.05/3 = 0.016*.

* *Difference between baseline and repeat score with a p < 0.05 (Wilcoxon 's signed rank test)*.

*UPDRS III, Unified Parkinson's Disease Rating Scale Part III; H&Y, Hoehn &Yahr Scale; FAB, Frontal Assessment Battery; a-WBAR, annual whole-brain atrophy rate*.

Clinical parameters were explored using the 18-item Movement Disorder Society-sponsored revision of the Unified Parkinson's Disease Rating Scale (MDS-UPDRS) motor symptoms (UPDRS III; Goetz et al., 2007) and the Hoehn and Yahr Scale (H&Y; Hoehn and Yahr, 1967), and executive function was assessed using the Frontal Assessment Battery (FAB; Dubois et al., 2000).

### MRI acquisition

Between 2012 and 2015, patients underwent an MRI brain scan. MRI images were acquired on a 3.0 T Philips Medical System. Axial T_1_-weighted images of the whole brain were obtained using a 3D inversion recovery prepared spoiled gradient echo (IR-SPGR) sequence. The following parameters were used: repetition time of 8.1 ms; echo time of 3.7 ms; inversion time of 450 ms; voxel size of 0.699 × 0.699 × 1 mm; excitation flip angle of 8°; matrix size of 248 × 226; field of view of 24 cm; and 198 axial slice of 1 mm. An experienced neuroradiologist (GG) assessed the MRI scans of every patient to rule out gross anatomical abnormalities. Patients underwent a second MRI brain scan at the time of the last study visit (12 months after the baseline scan). Subjects were included in the study if they had two MRI scans of adequate quality and the brain extraction step in SIENA functioned correctly. None of the MRI images included in this study showed any structural abnormalities other than atrophy-related changes. These inclusion criteria were assessed by a visual inspection of the raw and processed data for each patient scan. For both the baseline and follow-up assessments, the clinical data and MRI scans were acquired within 1 week of each other. The mean scan interval was 1.04 ± 0.07 years.

### Data analysis

All of the images were converted in NIFTI format using MRIcron software (http://people.cas.sc.edu/rorden/mricron/dcm2nii.html) in preparation for processing using SIENA. Before further processing, all of the data were anonymized by removing any reference to the patients' names from the image headers and ensuring that the file names were based on a unique ID rather than any of the patients' personal details, including their clinical group. The SIENA processing algorithm has been validated and described in detail elsewhere (Smith et al., 2002). Briefly, the processing stages are as follows: (1) Brain extraction (BET): segmentation of the brain from non-brain tissue for each scan, followed by skull extraction. (2) Registration: the segmented brain from the second (follow-up) scan is registered to that of the first (baseline) using a linear transformation. The two skull images are used as normalizing factors to constrain the scale and skew. (3) Tissue type segmentation: white matter and gray matter tissues are treated as one tissue and the cerebrospinal fluid as another. (4) Change analysis: detection of the brain edges on both registered brain images and then estimation of the motion of the brain surface edges. The direction of movement from the first image to second image indicates whether atrophy or growth has occurred. Finally, the percentage of global brain volume change is obtained for each subject from the mean of all of the edge point motions.

### Statistical analyses

Statistical analyses of the clinical data and clinical-imaging correlations were performed using the Statistical Package for Social Sciences (SPSS, Inc., Chicago, IL, USA, version 22). The results are presented as the mean ± SD. In all cases, a two-sided *p* < 0.05 was considered significant. Visual inspection of the data using histograms and QQ-plots was performed to test for violations of the assumption of a normal distribution. Levene's test of equal variances was used to verify the assumption of the homogeneity of variances. Because of these verifications, parametric and non-parametric statistical tests were used. One-way analysis of variance was performed for normally distributed data (age at examination, disease duration, a-WBAR). The Tukey test was used to control for multiple testing. Because disease severity and neuropsychological measures were non-normally distributed, between group differences were compared using Kruskal-Wallis tests, and when necessary, a *post hoc* procedure with Bonferroni correction for multiple tests (*p* = 0.05 was divided by 3) was used to compare the four disease groups. A χ^2^-test for homogeneity was used to compare the distribution of males and females across groups. The a-WBAR was calculated by dividing the WBAR values by the interscan interval in years. Clinical scores were also annualized by dividing the unit change between the assessments by years. Difference between baseline and repeat score were assessed using the Wilcoxon's signed rank test.

A-WBAR cut-off points for the differentiation between groups were determined by the Receiver Operating Characteristic curve (ROC) to define maximal sum of sensitivity and specificity.

### Standard protocol approval, registrations, and patient consent

Prior to inclusion, patients gave their informed written consent to participate in the study. The study was conducted according to International Standards of Good Clinical Practice (ICH guidelines and the Declaration of Helsinki). The project was approved by the local Research Ethics Committees of San Juan de Dios Hospital, Santiago, Chile.

## Results

### Demographics, clinical variables, and A-WBAR (Table 1)

There were no significant differences in age [IPD: 62.2 ± 11.5 (years ±SD); PSP 68.2 ± 6.3; MSA 60.4 ± 7.7], gender and disease duration between the IPD patients and both the MSA and PSP patients, although PSP patients were significantly older than MSA patients (*p* < 0.001). Disease duration was < 5 years for all groups. The MSA patients had a longer disease duration with a mean of 4.3 years [PSP 3.0 (*p* = 0.04)]. The PSP and MSA patients showed greater impairment on the H&Y scale than the IPD patients. The PSP patients showed greater impairment on the cognitive measures than the IPD and MSA patients.

MSA and PSP, but not IPD, showed significant mean deterioration over the follow-up period on a range of clinical measures.

**Figure 1:**
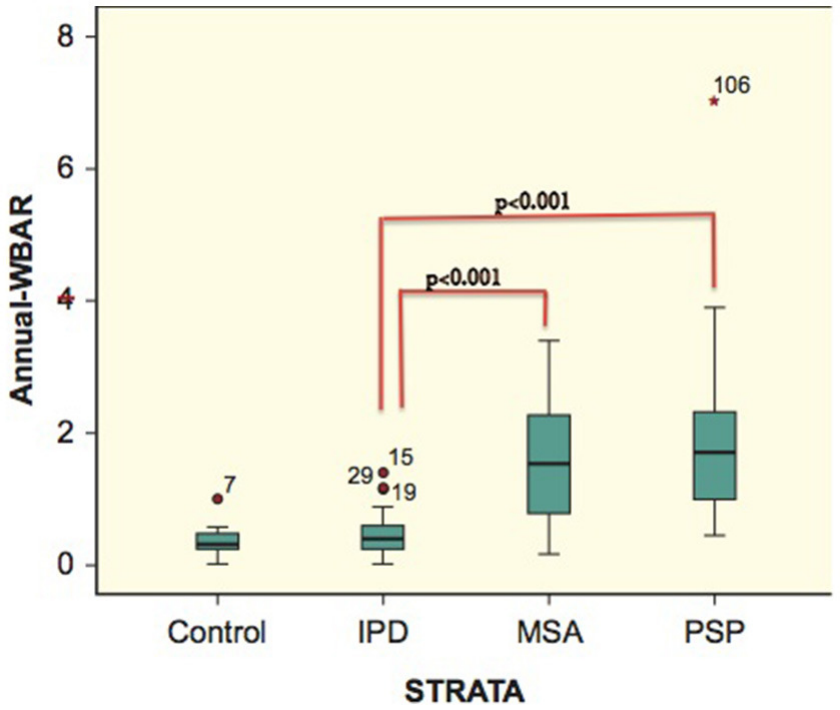
**Error bars showing 95% confident intervals (bars) of a-WBAR means for each group**. 7, 15, 29, 19, and 106 = outliers. STRATA = group.

**Figure 2:**
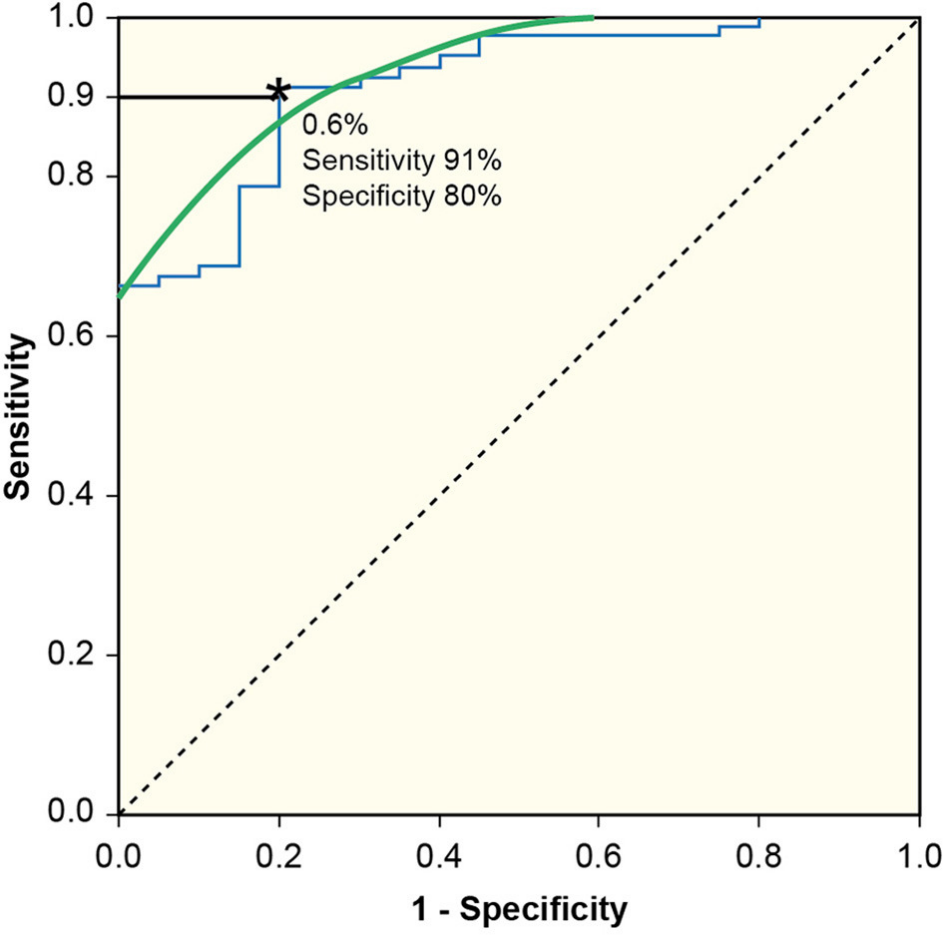
**Receiver Operating Characteristic curve (ROC) to define maximal sum of sensitivity and specificity**. a-WBAR at 0.6% is the cut-off point and differentiated patients with IPD from those with PSP and MSA with 91% sensitivity and 80% specificity.

In controls, the a-WBAR was 0.37% ± 0.28 (CI 95% 0.17–0.57), while in IPD patients a-WBAR was 0.54% ± 0.38 (CI 95% 0.32–0.68). The IPD patients did not differ from the controls. In PSP patients, the a-WBAR was 1.93% ± 1.1 (CI 95% 1.5–2.2). In MSA patients, a-WBAR was 1.65% ± 0.9 (CI 95%1.37–1.93). The MSA group did not differ from the PSP group. a-WBAR in the PSP and MSA groups was significantly higher than in the IPD group (*p* < 0.001; Figure 1). a-WBAR 0.6% differentiated patients with IPD from those with PSA and MSA with 91% sensitivity and 80% specificity (Figure 2); for IPD vs. MSA groups this value shows 85% sensitivity and 80% specificity, and for IPD vs. PSP groups 97% sensitivity and 75% specificity.

## Discussion

Diagnosis of Parkinsonian syndromes remains a difficult task that is based mainly on the clinical evaluation of neurologists, as no biological markers are currently available (Adler et al., 2014). Misdiagnosis not only means that patients may suffer from prognostic uncertainty but also means that clinical investigations are hampered by false positive cases. The inaccuracy in diagnosis is explained by the unknown *tempo* of widespread cellular destruction and the variable sites within the nigro-striatal dopaminergic system and/or cortices where neurodegeneration commences. A recent clinicopathologic study indicated that the clinical diagnostic capabilities for IPD have not advanced over the last 23 years (Rajput et al., 1991; Hughes et al., 1993; Adler et al., 2014), with only 26% accuracy for the clinical diagnosis of untreated or not clearly responsive patients, and 53% accuracy in patients who respond early to medications with disease duration < 5 years (Adler et al., 2014). An early longitudinal diagnostic biomarker to help to differentiate IPD vs. MSA and PSP is still a needed and was the main aim of this study.

In this study IPD patients did not show abnormal a-WBAR as was previously reported for IPD without dementia [0.6% (Paviour et al., 2006), 0.28% (Burton et al., 2005)]. We found an a-WBAR of 1.93% for PSP and 1.65% for MSA, which are higher than those in previous reports: approximately 1% for both PSP and MSA using BSI (Josephs et al., 2006; Paviour et al., 2006; Whitwell et al., 2007). Consistent with those reports, in the current study no significant difference was observed between a-WBAR in PSP and MSA (Paviour et al., 2006).

The a-WBAR reported in MSA and PSP are somewhat closer to those reported for Alzheimer disease using both BSI [2.1% (Schott et al., 2005), 2.37% (Chan et al., 2001), 2.78% (Fox and Freeborough, 1997)] and SIENA [1.9% (Sluimer et al., 2008b)]. It is plausible that cortical structures are the main contributors to whole brain atrophy in PSP and MSA. In PSP, neuronal loss is recognized in frontal, temporal, and limbic cortices and much less in parietal and occipital cortices (Verny et al., 1996). Such a neuronal loss is not considered to be typical in MSA. However, Papp and Lantos described high densities of glial cytoplasmic inclusions in the supplementary and primary motor cortical areas and subjacent white matter and moderate densities of glial cytoplasmic inclusions in the premotor area, cingulate motor area, and corpus callosum in MSA (Papp and Lantos, 1994). In a review of 203 proven MSA cases, some degree of cortical atrophy was observed in 21% of cases (Wenning et al., 1997), and *post mortem* examinations showed severe frontal atrophy (Inoue et al., 1997; Wakabayashi et al., 1998). *In vivo* data in MSA showed hypometabolism in motor, premotor and prefrontal cortices and parietal lobes (Kawai et al., 2008).A proton magnetic resonance spectroscopy study showed a significant reduction of N-acetylaspartate/creatine in the frontal cortex (Abe et al., 2000). Voxel-based morphometry studies have suggested that atrophy in the motor and prefrontal cortices are common findings in MSA (Brenneis et al., 2003).

By contrast, in levodopa-responsive IPD patients, evidence supports the idea that motor deficits are primarily related to the localized loss of selective dopaminergic neurons in the substantia nigra, with cortical and subcortical gray and white matter structures more preserved in comparison with those with PSP and MSA.

From a clinical perspective, an a-WBAR cutoff point of 0.6% may provide a potential retrospective application for a-WBAR to improve diagnostic accuracy (91% sensitivity and 80% specificity) for IPD vs. PSP and MSA, particularly in the initial stages when the clinical “plus syndrome” has not yet manifested and the response to levodopa treatment is being assessed.

With the current limited knowledge about the biology of MSA and PSP, interpretations and designs of MRI studies are mainly based on the information provided by proven cases (region of interest based studies). Sensitivity and specificity have been reported for many neuroimaging techniques based on region of interests for the differential diagnosis of IPD vs. MSA and PSP. Metabolic imaging using positron emission tomography (PET) studies of glucose metabolism were reported to have 86% sensitivity and 91% specificity to correctly categorize IPD from MSA and PSP (Hellwig et al., 2012). Dopamine transport (DAT) imaging using single photon emission CT (DAT-SPECT) is not efficient for the differentiation of IPD from PSP and MSA (Lokkegaard et al., 2002). Both molecular techniques PET and DAT-SPECT are expensive and not routinely available. A diffusion weighted imaging (DWI) study reported 90% sensitivity for differentiating PSP from IPD; however, in this study DWI was evaluated in only 10 PSP and 13 IPD patients (Seppi et al., 2003). Transcranial sonography has been reported to have 40% sensitivity and 61% specificity for the diagnosis of IPD (Bouwmans et al., 2013). Considering these data, a review concludes that no techniques are specifically recommended for routine use in clinical practice (Politis, 2014).

For disease-modifying treatments, the current challenge is to find biomarkers to accurately differentiate IPD from the aggressive MSA and PSP, early in the disease course. Ideally, MRI studies should also be based, as much as possible, on information obtained during the natural course of these diseases. The clinical and pathological aggressiveness of MSA and PSP may be due to global brain atrophy rather than degeneration of specific brain pathways and/or gray matter structures. a-WBAR within a normal range is unlikely to be observed in PSP or MSA but is likely to be observed in IPD patients. We propose a complementary use of clinical features (bradykinesia, rigidity, resting tremor, and response to dopaminergic drugs) and a-WBAR as a reasonable approach for the most accurate clinical diagnosis in these disorders early in the disease course.

A problem with using brain volume as a disease outcome is that it may not reflect physiologic or synaptic health. Furthermore, loss of brain volume might be influenced by causes that are common in people with chronic brain disorders, but only indirectly related to the disease itself, such as mild traumatic brain injury (MacKenzie et al., 2002), chronic alcohol abuse (Bartsch et al., 2007), nutritional deficiency, or hydration/dehydration (Kempton et al., 2009). However, these sources of variation are certainly less than that due to the disease itself.

As the current state of the art technique in neuroimaging, SIENA may be among the simplest MRI tools, but complex methodologies do not necessarily lead to robust and coherent results (Smith et al., 2004).

Overall, this study supports a complementary use of clinical tools and global rates of brain atrophy as an aid to clinical diagnosis between IPD vs. PSP and MSA.

## Author contributions

CG: Research project: Conception, Organization and Execution. Statistical Analysis: Design, Execution, Review and Critique. Manuscript: Writing of the first draft, Review and Critique. KB: Research project: Organization and Execution. Manuscript: Review and Critique. WS: Research project: Organization and Execution. Manuscript: Review and Critique. GG: Research project: Organization and Execution. Manuscript: Review and Critique. GF: Research project: Organization and Execution. Manuscript: Review and Critique.

## Funding

This study was supported by FONDECYT, grant 11121212, from the Chilean government and OAIC Hospital Clinico de la Universidad de Chile.

### Conflict of interest statement

The authors declare that the research was conducted in the absence of any commercial or financial relationships that could be construed as a potential conflict of interest.
